# Phylogeny and Fatty Acid Profiles of New *Pinnularia* (Bacillariophyta) Species from Soils of Vietnam

**DOI:** 10.3390/cells11152446

**Published:** 2022-08-07

**Authors:** Elena Kezlya, Yevhen Maltsev, Sergei Genkal, Zinaida Krivova, Maxim Kulikovskiy

**Affiliations:** 1 Laboratory of Molecular Systematics of Aquatic Plants, K.A. Timiryazev Institute of Plant Physiology RAS, IPP RAS, 127276 Moscow, Russia; 2 Papanin Institute for Biology of Inland Waters RAS, Borok, Nekouz, 152742 Yaroslavl, Russia

**Keywords:** 18S rDNA, biotechnology, diatoms, morphology, *rbc*L, taxonomy, ultrastructure

## Abstract

We studied the morphology, ultrastructure, and phylogeny of eight soil diatom strains assigned to the *Pinnularia* genus. Six of these strains, identified by us as new species, are described for the first time. We provide a comprehensive comparison with related species and include ecological data. Molecular phylogeny reconstruction using 18S rDNA and *rbc*L affiliates the new strains with different subclades within *Pinnularia*, including ‘borealis’, ‘grunowii’ and ‘stomatophora’. We also studied the fatty acid profiles in connection with the emerging biotechnological value of diatoms as a source of lipids. Stearic (36.0–64.4%), palmitic (20.1–30.4%), and palmitoleic (up to 20.8%) acids were the dominant fatty acids in the algae cultured on Waris-H + Si medium. High yields of saturated and monounsaturated fatty acids position the novel *Pinnularia* strains as a promising feedstock for biofuel production.

## 1. Introduction

Diatoms have been featured in multiple fields of technology and science, including paleoecological reconstructions [1], forensics [2], and biomedicine [3]. Strikingly, these microalgae produce about 20% of the primary biomass on Earth [4], while fixing about 25% of the global CO_2_. Moreover, diatoms accumulate enormous quantities of lipids—an estimated 10-fold compared with cultured terrestrial plants [5]. Despite the abundance of saturated and polyunsaturated long-chain fatty acids (FA), particularly eicosapentaenoic, docosahexaenoic, and arachidonic acids [4,6], FA profiles of diatoms remain understudied. The few reports are focused on marine species [7,8], notably *Phaeodactylum tricornutum* Bohlin rich in palmitic, palmitoleic, and eicosapentaenoic acids [7]. Another marine diatom, *Thalassiosira weissflogii* (Grunow) G.A. Fryxell et Hasle, is also rich in unsaturated palmitoleic and eicosapentaenoic acids [8]. Despite the eventual progress in FA studies on marine diatoms, similar studies focused on soil species are extremely rare. For instance, no FA profiles for such a ubiquitous genus as *Pinnularia* Ehrenberg 1843 can be found in the literature.

*Pinnularia* is one of the most numerous genera of biraphid diatoms [9]. A recent retrieval from Algaebase [10] includes 1432 specific and 1476 infraspecific epithets, of which 1403 have been flagged as accepted taxonomically. The *Pinnularia* algae are ubiquitously found in fresh waters and soils [11], reaching the highest diversity in the tropics (see the report on tropical diatoms of South America by Metzeltin and Lange-Bertalot [12]). *Pinnularia* is often mentioned as the most diverse group in soil algocenoses, as many of its species are cosmopolitan and common [13,14]. Numerous species of *Pinnularia* thrive in soils of spruce forests of the middle and southern taiga [15] and volcanic soils of Kamchatka, Russia [16]. Several *Pinnularia* species (*P. borealis*, *P. obscura*, *P. schoenfelderi*, and *P. sinistra*) have been identified as dominating taxa in the soils of Poland [17,18]. *P. obscura* and *P. schoenfelderi* also prevail in the Attert basin, Luxembourg [19], whereas *P. borealis*, followed by *P. appendiculata* and *P. microstauron*, prevail among soil crust diatoms in the arid Navajo National Monument, Arizona [20]. *Pinnularia* was also identified as the dominant diatom genus in paddy fields of Central Japan [21] and soils of the sub-Antarctic Crozet Archipelago [22]. Some species spread to aerophytic habitats, for instance, *P. issealana*, *P. obscura*, and notably, *P. borealis* inhabit a mossy overgrowth on white poplars (*Populus alba*) in south-eastern Poland [23].

*Pinnularia* phylogeny was comprehensively studied by several research groups [24,25,26,27]. Particular attention was paid to the cryptic diversity of the cosmopolitan terrestrial diatom *P. borealis* harboring eight molecular lineages [1,28,29]. Most thorough examinations of these lineages by light microscopy (LM) and scanning electron microscopy (SEM) failed to reveal morphological distinctions. The situation is rather typical and reflects the phylogenetic niche conservatism concept: cosmopolitan species often turn out to be a mixture of different strains/species, phylogenetically distinct but morphologically indistinguishable [30,31,32], which underscores the demand for the use of biochemical and molecular approaches in studying diatom evolution and their potential applications in biotechnology.

Comprehensive research on the diatoms inhabiting the moist tropical soils is still limited [33,34]. Studies on the algal flora of cultivated soils and semiaquatic lands often skip any molecular analysis [35,36,37] and thus tend to underrate the diversity, claiming, for instance, that paddy fields of northern Laos are dominated by *Nitzschia* sp. and *Pinnularia* sp. and the only *Pinnularia* species identifiable in cultivated soils of Egypt are *P. appendiculata* and *P. viridis*. A recent study on the algal flora of the unique flooded paddy land of Kuttanadu, South India, was focused exclusively on green algae. In Europe, molecular approaches to studying soil algae are also still uncommon, but arguably less ignored. One of the most extensive studies on the molecular diversity of green algae in soils was carried out in Germany [33] and used a cultivation approach to enhance the sensitivity. Molecular data for algal communities of tropical rainforests was published by the same research group in an article featuring green algae *Xylochloris* Neustupa, Eliás et Skaloud 2011 (clone C32U6-13) and *Jenufa* Nemcová, M. Eliás, Skaloud et Neustupa 2011 (clone S46L1-7) isolated from soil samples collected in the mountains of Ecuador (Cajanuma 3000 m a.s.l. and San Francisco 2000 m a.s.l.) [38].

Our research on soil algocenoses of the Cát Tiên National Park in South Vietnam started in 2019. The project has already yielded new diatom species *Mayamaea vientamica* [39] and *Placoneis cattiensis* [40]. This article covers morphology, ultrastructure, molecular phylogeny, and FA content for eight new strains of *Pinnularia* isolated from the tropical soils of Cát Tiên, six of them revealing strong distinctions indicative of new species nominated *P. minigibba*, *P. vietnamogibba*, *P. microgibba*, *P. insolita*, *P. ministomatophora*, and *P. paradubitabilis*.

## 2. Materials and Methods

**Figure 1:**
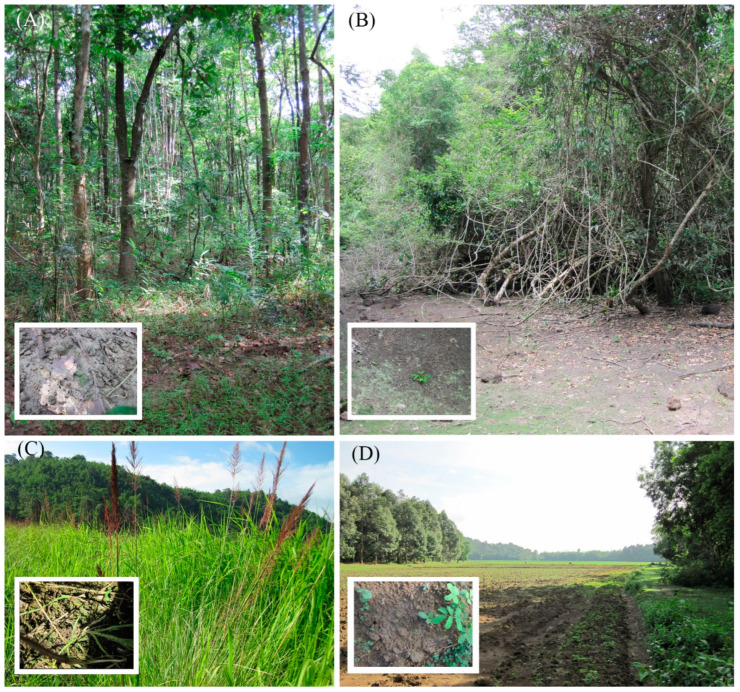
Sampling sites landscape in Cát Tiên National Park, Đồng Nai Province, Vietnam. (**A**) Forest (KT55). (**B**) The bottom of a dry reservoir (KT39). (**C**) Dry swamp (KT61). (**D**) Agricultural field (KT54).

Soil samples were collected in the Cát Tiên National Park, Đồng Nai Province, Vietnam, by E.S. Gusev and E.M. Kezlya in June 2019, and by D.A. Kapustin and N.A. Martynenko in March 2020. Sampling was during an expedition of the Joint Russian–Vietnamese Tropical Research and Technological Centre (the “Ecolan 3.2” Project). The strains were received from 5 samples taken in the forest (KT53, KT55), the bottom of a dry reservoir (KT39), dry swamp (KT61) and agricultural field (KT54) (Figure 1). The geographic position of samples and measured ecological parameters were indicated in Table 1.

The Cát Tiên National Park is located 150 km northeast of Ho Chi Minh City. The region belongs to the bioclimatic type of monsoon tropical climate with summer rains, relative humidity almost always exceeding 70%, and an average annual temperature of about 26 °C. From December to March there is almost no rainfall. The wet season peaks in August–September. At this time of the year, with up to 400–450 mm of precipitation per month, a significant part of the park becomes flooded. The main part of the territory is occupied by forests, which are of the monsoon, semi-deciduous type [41].

### 2.1. Sample Collection Procedure

The samples were collected as follows: the surface of the test site was examined to detect macrogrowth of algae, and then a composite sample was taken from an area of 10–30 m^2^. The composite sample consisted of 5–10 individual samples. For an individual sample, the topsoil was removed from an area of 5 to 20 cm^2^ with a metal scoop or shovel. After sampling, the instruments were cleaned and sterilized with ethanol. The samples were placed in labelled plastic zip bags and carried to the laboratory. The hot-drying method measured the absolute humidity [42] and the samples were air-dried and packaged.

To measure pH, we mixed 30 g of soil with 150 mL of distilled water [43]. The suspension was poured into a clean glass beaker and the measurements were performed with a Hanna Combo (HI 98129) device (Hanna Instruments, Inc., Woonsocket, RI, USA).

### 2.2. Culturing

Gathered materials were processed in the Laboratory of Molecular Systematics of Aquatic Plants of the Institute of Plant Physiology of the Russian Academy of Sciences (IPP RAS). A sample of soil was thoroughly mixed and placed into a Petri dish, then saturated with distilled water up to 60–80% of full moisture capacity and placed into an illuminated climate chamber. After a 10-day incubation, the sample was diluted with a small amount of distilled water, mixed gently, and the suspension was transferred to a Petri dish for LM using a Zeiss Axio Vert A1 inverted microscope. Algal cells were extracted with a micropipette, washed in 3–5 drops of sterile distilled water and placed into a 300 µL well on a plate for enzyme-linked immunoassay with Waris-H + Si [44]. Non-axenic unialgal cultures were maintained at 22–25 °C in a growth chamber with a 12:12 h light: dark photoperiod and checked every 10–14 days for 5 months.

For fatty acid analysis cultures were maintained on Waris-H + Si in 250 mL Erlenmeyer glass flasks with 150 mL medium, under constant orbital shaking (150 rpm in ELMI Sky Line Shaker S-3L, ELMI Ltd., Riga, Latvia) for 25 days at 25 °C. The light intensity was 100 μmol photons m^−2^ s^−1^ with a 16:8 h light/dark photoperiod. All analyses were performed in triplicate. Tables show the mean values and standard errors.

### 2.3. Microscopy

A culture was treated with 10% hydrochloric acid to remove carbonates and washed several times with deionized water for 12 h. Afterwards, the sample was boiled in concentrated hydrogen peroxide (≈37%) to remove organic matter. It was washed again with deionized water four times at 12 h intervals. After decanting and filling with deionized water up to 100 mL, the suspension was pipetted onto coverslips and left to dry at room temperature. Permanent diatom preparations were mounted in Naphrax. Light microscopic (LM) observations were performed with a Zeiss Axio Scope A1 microscope (Carl Zeiss Microscopy GmbH, Gottingen, Germany) equipped with an oil immersion objective (×100, n.a. 1.4, differential interference contrast) and Axiocam ERc 5s camera (Carl Zeiss NTS Ltd., Oberkochen, Germany). Valve ultrastructure was examined using a scanning electron microscope JSM-6510LV (IBIW; Institute for Biology of Inland Waters RAS, Borok, Russia). For scanning electron microscopy (SEM), part of the suspensions were fixed on aluminum stubs after air-drying. The stubs were sputter-coated with 50 nm of Au using an Eiko IB 3 machine (Eiko Engineering Co. Ltd., Tokyo, Japan). The suspension and slides are deposited in the collection of Maxim Kulikovskiy at the Herbarium of the Institute of Plant Physiology Russian Academy of Sciences, Moscow, Russia.

### 2.4. Molecular Study

Total DNA from the studied strains was extracted using Chelex 100 Chelating Resin, molecular biology grade (Bio-Rad Laboratories, Hercules, CA, USA), according to the manufacturer’s protocol 2.2. Partial 18S rDNA (395–400 bp, including the highly variable V4 region of the 18S gene), and partial *rbc*L plastid genes (921–957 bp) were amplified using primers D512for and D978rev from Zimmermann et al. [45] for 18S rDNA fragments and *rbc*L404+ from Ruck and Theriot [46] and *rbc*L1255- rom Alverson et al. [47] for *rbc*L fragments.

Amplifications were carried out using premade polymerase chain reaction (PCR) mastermixes (ScreenMix by Evrogen, Moscow, Russia). Amplification conditions for the 18S rDNA gene were as follows: initial denaturation for 5 min at 95 °C followed by 35 cycles of 30 s denaturation at 94 °C, 30 s annealing at 52 °C, and 50 s extension at 72 °C, with the final extension for 10 min at 72 °C. Amplification conditions for the *rbc*L gene were as follows: initial denaturation for 5 min at 95 °C followed by 45 cycles of 30 s denaturation at 94 °C, 30 s annealing at 59 °C, and 80 s extension at 72 °C, with the final extension for 10 min at 72 °C. PCR products were visualized by horizontal electrophoresis in 1.0% agarose gel stained with SYBR^TM^ Safe (Life Technologies, Carlsbad, CA, USA). The products were purified with a mixture of FastAP, 10×FastAP Buffer, Exonuclease I (Thermo Fisher Scientific, Waltham, MA, USA), and water. The sequencing was performed using a Genetic Analyzer 3500 instrument (Applied Biosystems, Waltham, MA, USA).

**Figure 2:**
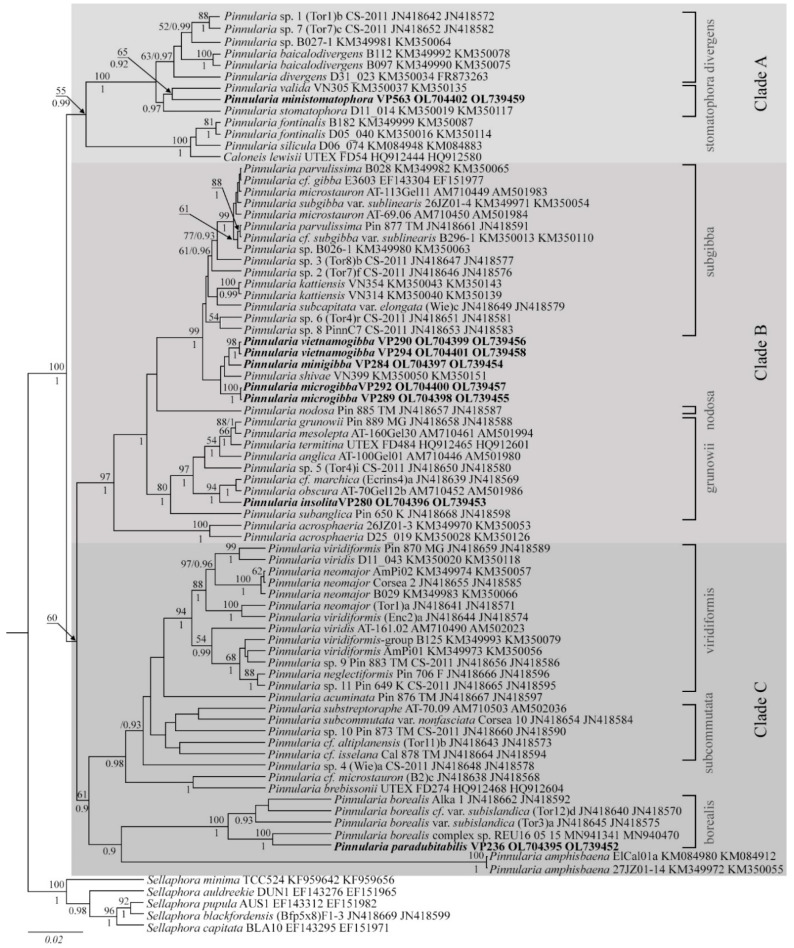
Phylogenetic position of the new *Pinnularia* species (indicated in bold) based on Bayesian inference for the partial 18S rDNA and *rbc*L genes. The total length of the alignment is 1407 characters. Bootstrap supports from ML (constructed by RAxML) and posterior probabilities from BI (constructed by Beast) are presented on the nodes in order. Only BS and PP above 50 and 0.9 are shown. Strain numbers (if available) and GenBank numbers are indicated for all sequences.

Editing and assembling of the consensus sequences were carried out by processing the direct and reverse chromatograms in Ridom TraceEdit ver. 1.1.0 (Ridom GmbH, Münster, Germany) and Mega7 software [48]. The reads were included in the alignments along with corresponding sequences of 72 diatom species downloaded from GenBank (taxa names and Accession Numbers are given in Figure 2). Three centric diatom species were chosen as the outgroups.

The nucleotide sequences of the 18S rDNA and *rbc*L genes were aligned separately using the Mafft ver. 7 software (RIMD, Osaka Japan) and the E-INS-i model [49]. The final alignments were then carried out: unpaired sites were visually determined and removed from the beginning and the end of the resulting matrices. For the protein-coding sequences of the *rbc*L gene, we checked that the beginning of the aligned matrix corresponds to the first position of the codon (triplet). The resulting alignments had lengths of 450 (18S rDNA) and 957 (*rbc*L) characters. After removal of the unpaired regions, the aligned 18S rDNA gene sequences were combined with the *rbc*L gene sequences into a single matrix Mega7 (Appendix S1).

The data set was analyzed using the Bayesian inference (BI) method implemented in Beast ver. 1.10.1 software (BEAST Developers, Auckland, New Zealand) [50] to construct a phylogeny. For the alignment partition the most appropriate substitution model, shape parameter α and a proportion of invariable sites (pinvar) were estimated using the Bayesian information criterion (BIC) as implemented in jModelTest ver. 2.1.10 (Vigo, Spain) [51]. This BIC-based model selection procedure selected the following models, shape parameter α and a proportion of invariable sites (pinvar): HKY + I + G, α = 0.6250 and pinvar = 0.5170 for 18S rDNA; TPM1uf + I + G, α = 1.1070, and pinvar = 0.7470 for the first codon position of the *rbc*L gene; JC + I + G, α = 0.3830 and pinvar = 0.7320 for the second codon position of the *rbc*L gene; TPM2uf + G and α = 0.5470 for the third codon position of the *rbc*L gene.

We used the F81 model of nucleotide substitution instead of TPM1uf, the HKY model instead of JC, and TPM2uf, given that they were the best matching model available for BI. A Yule process tree prior was used as a speciation model. The analysis ran for 5 million generations with chain sampling every 1000 generations. The parameters-estimated convergence, effective sample size (ESS), and burn-in period were checked using the Tracer ver. 1.7.1 software (MCMC Trace Analysis Tool, Edinburgh, United Kingdom). [50]. The initial 25% of the trees were removed, and the rest were retained to reconstruct a final phylogeny. The phylogenetic tree and posterior probabilities of its branching were obtained based on the remaining trees, having stable estimates of the parameter models of nucleotide substitutions and likelihood. The Maximum Likelihood (ML) analysis was performed using RAxML software [52]. The nonparametric bootstrap analysis with 1000 replicas was used. The phylogenetic tree topology is available online in Appendix S2. FigTree ver. 1.4.4 (University of Edinburgh, Edinburgh, United Kingdom) and Adobe Photoshop CC ver. 19.0 software (Adobe, San Jose, CA, USA) were used for viewing and editing the trees.

### 2.5. Fatty Acid Analysis

Biomass preparation for determining the fatty acid methyl ester (FAME) profiles was performed according to Maltsev et al. [53]. The diatom suspensions were conveyed to 15–50 mL tubes (depending on the volume). The cells were pelleted at room temperature for 3 min at 3600 g. The supernatant was removed, and the pelleted cells were resuspended in 10–15 mL (depending on the amount of biomass) of distilled water, quantitatively transferred to 15 mL centrifuge tubes, and pelleted again by centrifugation. The supernatant was removed, and samples were quantitatively transferred to a 50 mL round-bottom flask. Heptadecanoic acid (Sigma-Aldrich, St. Louis, MO, USA) was used as the internal standard for the fatty acid composition determination. To avoid the oxidation of unsaturated fatty acids, all samples were processed under an argon atmosphere. Ten milliliters of a 1 M solution of KOH in 80% aqueous ethanol was added to the dry residue, and the flask was sealed with a reflux condenser, and kept for 60 min at the boiling point of the mixture (~80 °C). After the time-lapse, the solvents were evaporated in vacuo to a volume of ~3 mL and quantitatively transferred with distilled water to a 50 mL centrifuge tube to a total volume of 25 mL, followed by extracting the unsaponifiable components with 10 mL portions of *n*-hexane (Himmed, Moscow, Russia) 3 times. To accelerate the separation of the phases, the tube was centrifuged for 5 min at room temperature and 2022× *g*. After that, the aqueous phase was acidified to a slightly acidic reaction (on indicator paper) with a few drops of 20% sulfuric acid (Himmed, Moscow, Russia), and free fatty acids were extracted with 20 mL of *n*-hexane. The hexane solution of free fatty acids was transferred to a dry 50 mL round-bottom flask, and the solvent was evaporated to dryness using a rotary evaporator IKA RV-10 (IKA-WERKE, Staufen im Breisgau, Germany), after which 10 mL of absolute methanol (Sigma-Aldrich, St. Louis, MO, USA) and 1 mL of acetyl chloride (Sigma-Aldrich, St. Louis, MO, USA) were added to the dry residue. The flask, closed with a reflux condenser, was kept for one hour at 70 °C, then the solvents were evaporated to dryness, a few drops of distilled water were added to the dry residue, and FAMEs were extracted with *n*-hexane.

The obtained FAMEs were analyzed using an Agilent 7890A gas-liquid chromatograph (Agilent Technologies, Santa Clara, CA, USA) with an Agilent 5975C mass spectrometric detector. A DB-23 capillary column 60 m long and 0.25 mm in diameter was used (Agilent Technologies, Santa Clara, CA, USA). The remaining conditions of the analysis were as follows: carrier gas was helium, flow rate of 1 mL min^−1^, 1 μL volume of injected sample, 1:5 flow divider, and the evaporation temperature of 260 °C. Temperature gradient program: from 130 to 170 °C in 6.5 °C min^−1^ steps; from 170 to 215 °C in 2.5 °C min^−1^ increments, 215 °C for 25 min, from 215 to 240 °C in 40 °C min^−1^ increments, and the final stage lasting 50 min at 240 °C. The operating temperature of the mass spectrometric detector was 240 °C and the ionization energy was 70 eV.

## 3. Results and Discussion

*Pinnularia* specimens are ubiquitously found in soil samples collected in the Cát Tiên National Park. Based on the results of DNA sequencing, LM/SEM observations, and FA profiling of eight different strains of *Pinnularia*, we regard six of them as new species. For the sake of clarity, we address these strains by specific epithets ahead of the formal description.

### 3.1. Molecular Phylogeny

Comprehensive studies on *Pinnularia* phylogeny [24,25] confirm its monophyletic origin; the genus splits into three clades stably supported by the analysis. In a study by Souffreau et al. [25], who used five genetic markers—two nuclear (18S rDNA and 28S rDNA), two plastid (*rbc*L and *psb*A), and a mitochondrial cox1, these clades were designated A, B, and C. Each clade consists of several subclades characterized by morphological similarity about shapes (valves and apices, raphe endings linear or rounded, raphe fissures straight or undulate, chloroplasts H-shaped or elongated, with pyrenoids or not, etc.) and specific markings (ghost striae, fascia, wart-like bodies, etc.). Although our phylogenetic analysis involved only two markers (nuclear 18S rDNA and plastid *rbc*L), it perfectly preserved the tree topology and maintained high support to all clades and subclades introduced by Souffreau et al. [25]. The tree was expanded through the addition of the new taxa, which showed full morphological consistency with their parental subclades (Figure 2).

Clade A includes two previously characterized subclades exemplified by *Caloneis* Cleve 1894 and *P.* cf. *divergens*. Here we complement the ‘divergens’ subclade with new species; a morphological feature is a fascia with rounded thickenings at the margin. Furthermore, the expansion of *Pinnularia* phylogeny revealed a new subclade of clade A, ‘stomatophora’, comprising *P. ministomatophora*, *P. valida* (strain VN305), and *P. stomatophora* (strain D11_014), all of them presenting with characteristic hollow markings on the external surface of the valve, crescent-shaped (*P. stomatophora* (p. 456, pl. 98, Figure 8 of [11]) or irregular (*P. ministomatophora* sp. nov.).

Clade B includes three subclades, ‘grunowii’, ‘nodosa’, and ‘subgibba’, of diminutive linear algae with bulbous apices and rounded external endings of the raphe [25]. Our phylogeny investigation recognizes these subclades with high support. However, the two species of ‘nodosa’ subclade, *P. nodosa* and *P. acrosphaeria* [25] split into distinct branches (Figure 2). These species also show morphological distinctions: *P. acrosphaeria* display mottled, structured areas on both outer and inner surfaces of the valve (p. 296, pl. 19, Figures 1–6 of [11]) whereas in *P. nodosa* with the valve is smooth on the inside and its entire outer surface is heavily structured (p. 307, pl. 24, Figures 1–6 of [11]).

A morphological feature of the ‘subgibba’ subclade is ghost striae in the central area [25,26,27]. The term ghost striae was proposed by Cox [54] and refers to thinnings on the inner surface of the valve, corresponding in size and spacing to normal striae. It should be noted that there is no unified name for these structures defined as “large markings, differently structured on both sides and larger in the ventral side” [11], “deepenings on the inside of the valve” [11] or “depressions in the central area” [55]. Our phylogenetic study encompassed *P. microstauron*, *P. parvulissima*, *P.* cf. *gibba*, *P.* cf. *subgibba* var. *sublinearis*, *P. subcapitata* var. *elongata*, *P. kattiensis*, and several strains of *Pinnularia* sp. The newly identified soil species *P. microgibba*, *P. minigibba*, and *P. vietnamogibba* along with *P. shivae* form a separate branch within the ‘subgibba’ subclade (Figure 2). Ultrastructural examination of *P. microgibba*, *P. minigibba*, and *P. vietnamogibba* specimens by SEM revealed ghost striae in the central area for all of them, consistently with their phylogenetic affiliation.

Incidentally, the ‘grunowii’ subclade also splits into two branches, which happen to have a different ultrastructure of the central area (Figures 2 and 10 and Figures in Krammer [11], Jahn and Kusber [56]). Species with ghost striae, namely *P. anglica P. grunowii*, and *P. mesolepta* (p. 423, pl. 82, Figures 7 and 8 of [11,56]), are phylogenetically divergent from the diminutive *P. marchica*, *P. obscura* (scanning images are available in Jahn and Kusber [56]), and *P. insolita* sp. nov. with no markings in the central field.

Clade C specimens have no markings in the central area but exhibit wide strokes on the valves. Our study generally preserved the topology for this clade introduced by Souffreau et al. [25], while the ‘borealis’ subclade was complemented by new strains and new species *P. paradubitabilis*. Sister lineages to the ‘borealis’ subclade are constituted by strains of *P. amphisbaena* and two related subclades, ‘subcommutata’ and ‘viridiformis’, comprising large specimens of linear-elliptical shape with almost parallel striae and small central areas, undulate external raphe fissures and with linear (for subcommutata) or rounded (for viridiformis) central raphe endings. In contrast with the original version [25], *P*. cf. *microstauron*, *P*. *brebissonii*, *P. accuminata*, *Pinnularia* sp. 4 (Wie)a, and strains morphologically similar to *P*. *microstauron* split into separate branches within clade C (Figure 2).

It is interesting to observe that certain morphological distinctions, notably the presence of ultrastructural surface markings in the central area, consistently follow phylogenetic clades. Species presenting with markings in the central area can be reliably classified on their basis as ‘divergens’, ‘stomatophora’, ‘subgibba’, ‘nodosa’, and ‘gronowii’ groups.

### 3.2. Comparative Morphology

*Pinnularia minigibba* sp. nov. is similar to several species of the *P. gibba* complex (Table 2). The new species must not be confused with *P. australogibba* var. *subcapitata* [55]. The similarities include the outlines and proportions of the valves, characteristic shape of the central area with a broad fascia, and ghost striae. However, *P. minigibba* has wider valves (7–8 μm vs. 5.7–7.3 μm in *P. australogibba* var. *subcapitata*) with slightly concave margins (as opposed to convex sides of *P. australogibba* var. *subcapitata*). Other differences include stria density (respectively, 9–10 in 10 μm vs. 11–12 in 10 μm) and the shape of axial area (≤1/4 of the width and slightly wider towards the midportion in *P. minigibba* vs. lanceolate in *P. australogibba* var. *subcapitata*); also, in the new species, the stria is more tilted in the midportion. Another closely similar species, *P. parvulissima* (p. 397, pl. 69, Figures 6–11 of [11]), can be differentiated by larger size (34–70 μm length to 10–12 μm width vs., respectively, 40–43 to 7–8 in *P. minigibba*); besides, *P. parvulissima* have convex margins and wider axial area. The similarity of *P. minigibba* with certain strains of the polymorphic widespread *P. microstauron* (Ehrenberg) Cleve should be noted as well. For instance, it can be confused with *P. microstauron* var. *angusta* (p. 361, pl. 51, Figures 4–7 of [11]), which is a smaller variety with wider fascia, and a particularly challenging specimen shown in Figure 18, pl. 164 of Metzeltin et al. [57]. About these examples, *P. minigibba* can be differentiated by stria density (9–10 in 10 μm vs. 10–12 in 10 μm in *P. microstauron* var. *angusta* and 11 in 10 μm in *P. microstauron* sensu Metzeltin et al. [57], as well as concave margins (vs. parallel or subtly convex in *P. microstauron*), and characteristic presence of ghost striae in the central area. Despite the apparent absence of ghost striae in LM images of *P. microstauron* var. *angusta* and *P. microstauron* sensu Metzeltin et al. [57], ultrastructural examinations of archetypal *P. microstauron* specimens (e.g., AT_112Gel04, AT_113Gel11 cultures [55,56]) reveal hollowed markings on the inner surface of the valve confined to the central area, corresponding to ghost striae, consistently with the assignment to the ‘subgibba’ subclade (p. 371, Figure 3 of [26]).

*Pinnularia vietnamogibba* sp. nov. must not be confused with two closely similar taxa—*P. gibba* var. *subsancta* (pl. 13, Figure 7 of [58]; p. 521, pl. 140, Figures 9 and 10 of [59]) and *P. australogibba* (p. 215, Figures 94–101 of [55]). According to Krammer [11] (P. 111), most specimens defined in literature as *P. gibba* var. *sancta* (Grunow) Meister arguably belong to a smaller variety described by Manguin (pl. 13, Figure 7 of [58]). According to Hustedt [60], this taxon is widespread in the tropics, whereas *Pinnularia* *australogibba* is found in ravines at Point Del Cano, Île Amsterdam in the southern Indian Ocean. The similarities of these species with the newly identified *P. vietnamogibba* include the shape of the central area with a broad fascia and ghost striae. However, *P. gibba* var. *subsancta* and *P. australogibba* specimens have lanceolate outlines, whereas *P. vietnamogibba* are linear, with the margins parallel or subtly bulging. *P. vietnamogibba* also has much broader fascia, which constitutes 17–20% of the valve length (cf. 4–10.3% of the length in *P. gibba* var. *subsancta* or 7.7–14.5% in *P. australogibba*) and lower stria density (10–11 in 10 μm vs. 13–15 in 10 μm and 12–13 in 10 μm in *P. gibba* var. *subsancta* and *P. australogibba*, respectively). *P. vietnamogibba* can also be confused with the already mentioned *P. microstauron* var. *angusta* (p. 361, pl. 51, Figure 4 of [11]; p. 81, Figures 47–49 of [61]), which may reach a similar size and have similar stria densities and similar shapes of the central and axial areas. However, in *microstauron*-like morphologies, the ends are distinctly offset and much smaller compared with the width of the valve, in contrast to *P. vietnamogibba*. Another morphometrically similar species, *P. tagliaventiae* (p. 417, pl. 130, Figures 1–7 of [62]), can be distinguished by triangulate sides, whereas the sides of *P. vietnamogibba* are straight.

*Pinnularia microgibba* sp. nov. phylogenetically and morphologically closely related with ‘subgibba’-group species [25,26], particularly with strains *Pinnularia* sp. 6 (Tor4)r and *Pinnularia* sp. 3 (Tor8)b (from littoral zones of freshwater bodies in Chile, see Table 2). These strains are virtually identical in size, shape, and outline; the only clue to their morphological distinction is stria density (12 in 10 μm. for (Tor8)b and 14 in 10 μm for (Tor4)r). Meanwhile, they are phylogenetically independent (Figure 2). Morphometric data of *P. microgibba* almost totally match those of (Tor4)r and (Tor8)b, except for the shape the of central area: linear, broadening towards the valve margins in (Tor4)r and (Tor8)b, and rhombic in the new species. Phylogenetically, the strains of *P. microgibba* together with *P. minigibba* and *P. vietnamogibba* form a separate branch within the ‘subgibba’ subclade.

It should be kept in mind that ghost striae are often poorly distinguishable in light micrographs. For this reason, *P. microgibba* can be easily confused with certain diminutive species with narrow linear outlines and broad fascia, including the widespread, ubiquitous, and cosmopolitan *P. sinistra* [11,63]. However, *P. microgibba* has concave margins and a rhombic central area with ghost striae, whereas *P. sinistra* has a linear central area without markings (p. 265, Taf. 37, Figure 16 of [63]). Interestingly, a population of *P. sinistra* inhabiting the small oceanic island Île Amsterdam, situated in the southern part of the Indian Ocean (p. 225, Figures 194–209 of [55]), also has concave margins and rhombic central area; however, these taxa can be distinguished from both the type of *P. sinistra* and type of *P. microgibba* by their relatively wide lanceolate axial area (compared with the narrow-linear axial area in type populations of *P. sinistra* and *P. microgibba*). Several images for certain species found in the literature can be confused with *P. microgibba* as the distinctions are very subtle and demand close attention (Table 2). For instance, the valves of *P. microstauron* var. *angusta* (p. 361, pl. 51, Figures 5 and 6 of [11]) are wider (6.5–8.0 μm) compared with *P. microgibba* (5.5–6 μm). *P. subcapitata* W. Gregory (given as *P. hilseana* Janisch 1861 (p. 757, pl. 205, Figure 9 of [64]) and *P*. cf. *hilseana*/*P*. cf. *fotii* Bily et Marvan (p. 757, pl. 205, Figures 26 and 27 of [64]) have distinctly capitate valve ends (apices) compared with the subcapitate apices in *P. microgibba*. *Pinnularia* cf. *saprophila* (p. 573, pl. 164, Figures 3 and 4 of [57]) have a lower stria density (9.5–11.0 in 10 μm) compared with *P. microgibba* (11.0–12.0 in 10 μm). *Pinnularia pisciculus* found in low nutrient waters, mosses, and dry soils from India (pl. 73 [65]) display characteristic triundulate sides and capitate apices. *Pinnularia similiformis* var. *koreana* (p. 291, pl. 16, Figures 3–6 of [11]) is lengthy (40–60 μm), whereas *P. microgibba* is 36–40 μm. *Pinnularia marchica* (p. 283, pl. 12, Figures 11–17 of [11]) have elongated rostrate apices different from the subcapitate apices of *P. microgibba*. Ghost striae in the central area represent an important identifier; however, it should be applied with caution, as SEM data are not universally available and the sensitivity of LM about ghost striae is limited (Table 2).

*Pinnularia insolita* sp. nov. can be recognized by its unusual outline with concave side margins and narrowed apices, as well as the characteristic shape of the central area with a prominent fascia broadening towards the margins. Nevertheless, certain varieties of other *Pinnularia* species are similar (Table 2). Large specimens of *P. pisciculus* Ehrenberg (p. 429, pl. 85, Figures 25 and 26 of [11]) are similar to *P. insolita* (length 22–50 μm, width 6.0–8.3 μm in *P. pisciculus* vs. length 50–52 μm and width 7–7.5 μm in *P. insolita***)**, stria density (10.5–12.0 μm in 10 μm vs. 11–12 in *P. insolita*), and overall outline. Nevertheless, the algae can be reliably differentiated by their apices shape (capitate in *P. pisciculus* and subtly rostrate in *P. insolita*) and the shape of the central area (rhombic in *P. pisciculus* vs. broadening towards the margins in *P. insolita*). Moreover, larger specimens of *P. pisciculus* tend to have subtly triundulate side margins, whereas the sides of *P. insolita* are concave. Another similar species with *P. insolita* is *P. brebissonii* var. *bicuneata*, whose largest annotated specimen (p. 351, pl. 46, Figure 9 of [11]) resembles *P. insolita* by its size, shapes of the valve apices and central area, and stria density. However, in *P. brebissonii* var. *bicuneata* the sides are straight and parallel (vs. subtly concave in *P. insolita*), the valves are wider (8–11 μm vs. 7–7.5 μm in *P. insolita*), and the axial area is narrow (vs. moderate, about 1/3 width of the width, in *P. insolita*). Other confusing specimens are those of *P. cavancinii* (p. 411, pl. 127, Figures 1–3 of [57]), which also have narrowed apices, as well as valve dimensions, stria densities, and central/axial areas similar to *P. insolita*. However, these can also be differentiated by the outline (rhombic-lanceolate to elliptic-lanceolate in *P. cavancinii* vs. linear with subtly concave sides in *P. insolita*). Molecular phylogeny affiliates *P. insolita* with the subclade of *P. obscura* AT_70Gel12b and *P. marchica* Ecrins4_a. Voucher images available for these strains correspond to the species descriptions [24,25,56] and show considerable morphometric differences with *P. insolita*, including the smaller size (length ≤ 37 μm and width 6.3 μm vs. 50–52 μm and 7–7.5 μm in *P. insolita*) as well as distinctive outline and stria densities (Table 2). It should be noticed, however, that a common morphological feature of this subclade is the absence of any markings on both sides of the valve within the central area (Table 2).

*P. ministomatophora* sp. nov. occupies a distinct position in the phylogenetic tree (Figure 2), sharing the ‘stomatophora’ subclade with *P. valida* (strain VN305) and *P. stomatophora* (strain D11_014). One reliable morphometric distinction is the size: *P. valida* and *P. stomatophora* are larger (120 μm and 55–115 μm in length, respectively, vs. 44–57 μm for *P. ministomatophora*). Still, by both the size and the characteristic hollow markings on the outer surface of the valve within the central area, *P. ministomatophora* can be confused with *P. stomatophora* var. *irregularis* Krammer (p. 460, pl. 101, Figures 4–10 of [11]). The last species was isolated from the aerophytic habitats of Bavaria. Nevertheless, these species can be distinguished morphologically by outlines (*P. ministomatophora* specimens have convex sides and subcapitate apices, in *P. stomatophora* var. *irregularis* have smooth outlines with parallel margins and apices rounded), raphe morphology (outer fissures curved in *P. stomatophora* var. *irregularis* and straight in *P. ministomatophora*), and the shape of the central area (fascia significantly broader in *P. ministomatophora*); besides, *P. ministomatophora* are narrower (width 7–9.5 μm vs. 10–11 μm *P. stomatophora* var. *irregularis*). Apart from that, *P. ministomatophora* may resemble certain specimens of *P. graciloides* var. *triundulata* (p. 461, pl. 101, Figures 2 and 3 and p. 457, pl. 99, Figure 100 (SEM) of [11]; p. 635, pl. 88, Figures 10 and 11 of [9]) with dimensions towards the lower end of the size range and muted undulations; however, the valves of *P. ministomatophora* are significantly narrower (width 7.5–9.5 μm vs. 11–13 μm in *P. graciloides* var. *triundulata*). It can also be confused with certain representatives of the ‘subgibba’ subclade, including *P. parvulissima* (p. 397, pl. 69, Figures 8 and 9 of [11]), *P. subgibba* var. *undulata* (p. 387, pl. 64, Figure 7 of [11]), *P. pseudogibba* (p. 392, pl. 67, Figures 8–11 of [11]), and *P. vietnamogibba* sp. nov. However, *P. parvulissima* are significantly wider (10–12 μm vs. 7.5–8 μm), *P. subgibba* var. *undulata* have triundulate side margins, *P. pseudogibba* differ by the shape of the central area (no broad fascia) and *P. vietnamogibba* differ by apical shapes (*P. ministomatophora* have subcapitate apices whereas in *P. vietnamogibba* the apices are smoothly rounded) and the shape of the central area (*P. vietnamogibba* have wider fascia). Also, of course, *P. vietnamogibba* presents with ghost striae, i.e., depressions on the inside of the valve within the central area (p. 403, pl. 72, Figures 3 and 5 of [11]) whereas *P. ministomatophora* have similar depressions on the outer surface.

*Pinnularia paradubitabilis* sp. nov. occupies a separate phylogenetic position within the ‘borealis’ subclade and at a first glance appears similar to archetypal specimens of this ambiguous taxonomic group. Morphology and phylogeny of the widespread polymorphic *P. borealis* have been extensively described in several studies, with multiple morphotypes illustrated [1,11,28,29,67]. However, despite the overall semblance, the new species can be convincingly distinguished from the rest of ‘borealis’ by its broad fascia. In the opinion of Kollár et al. [26], the breadth of fascia represents a more stable indicator than valve outlines or aspect ratio. None of *P. borealis* morphotypes have fascia, other than the 1–2 stria missing in the central area. In addition, according to the comprehensive description by Krammer [11], *P. borealis* have wider valves (8.5–10.0 μm vs. 6–7 μm in *P. paradubitabilis*). The outlines are different as well: in *P. borealis* valves are linear or elliptic-linear with moderately convex margins, ends rounded, whereas in *P. paradubitabilis* the valves are linear with margins parallel or subtly concave, ends bluntly rounded. By morphometric features including size, outline, fascia, and stria density (Table 2), the newly identified species is most similar to *P. dubitabilis* Hustedt found in Java and Sumatra (p. 276, pl. 9, Figures 7–9 of [11]). On the other hand, *P. dubitabilis* have very short striae, confined to the narrow space along margins, and a wide axial area, whereas *P. paradubitabilis* show the opposite patterns with extensive striae and narrow axial area. Several other species from the section Distantes (Cleve) Patrick are similar to our new taxon, most notably those with fascia: *P. angustiborealis* Krammer et Lange-Bertalot and *P. intermedia* (Lagerstedt) Cleve (Table 2). However, *P. angustiborealis* have moderately convex side margins (cf. parallel or concave margins of *P. paradubitabilis*) and the width of 7.4–8.0 μm (cf. 6–7 μm of *P. paradubitabilis*). Comparison with *P. intermedia* reveals clear differences in stria density (7–10 in 10 μm vs. 5–6 in 10 μm in *P. paradubitabilis*) and apices shape (capitate in *P. intermedia* vs. obtusely rounded in *P. paradubitabilis*). The new species can also be confused with *P. angulosa*; however, *P. angulosa* have larger valves (42–53 μm length and 9.7–10.3 μm width vs., respectively, 39–41 μm and 6–7 μm in *P. paradubitabilis*) with the wide axial area (cf. the narrow axial area in *P. paradubitabilis*).

### 3.3. Fatty Acid Profiles

A study on FA content of the identified *Pinnularia* strains is all the more valuable given the general scarcity of biochemical research on soil diatoms. Analysis of the biomass at the stationary phase of growth revealed the domination of saturated FA, notably 16:0 palmitic acid (within the range of 20.1–30.4% of total FA) and 18:0 stearic acid (36.0–64.4%), for all strains (Table 3). In addition, *P. insolita* VP280, *P. microgibba* VP292, *P. paradubitabilis* VP236, *P. vietnamogibba* VP294, *P. ministomatophora* VP563 accumulated high amounts of monounsaturated 16:1n-7 palmitoleic acid (15.2–20.8%). Small amounts of saturated 20:0 arachidic acid (≤0.4%) were determined in all strains, whereas *P. vietnamogibba* VP294, *P. minigibba* VP284, and *P. paradubitabilis* VP236 also produced small amounts of the long-chain saturated 22:0 behenic acid (≤0.2%). The long-chain omega-3 polyunsaturated 20:5n-3 eicosapentaenoic acid (1.1%) was determined exclusively in *P. insolita* VP280 biomass. Low concentrations of the omega-6 20:4n-6 arachidonic acid (0.5–2.3%) were detected in *P. insolita* VP280, *P. ministomatophora* VP563, *P. minigibba* VP284, *P. microgibba* VP289 and VP292 biomasses. The highest fatty acid yields in dry biomass were obtained for *P*. *minigibba* and strain *P*. *vietnamogibba* VP290 in amounts up to 47.8 mg g^–1^ (Table 3). Similar values were obtained for strains *Sellaphora pupula* FC2 grown in the air (54.83 mg g^–1^) [68] and *Entomoneis* cf. *paludosa* 8.0727-B on the stationary growth phase (39 mg g^–1^) [69]. Yields were the lowest for *P*. *insolita* and *P*. *paradubitabilis* strains (Table 3).

These data were compared with the published evidence on FA profiles of another soil diatom, *Nitzschia palea* SAG 1052-3a [70], also dominated by saturated and monounsaturated acids. In *Nitzschia palea* SAG 1052-3a, about three quarters of total FA were constituted by equal proportions of saturated 14:0 myristic (26%), saturated 16:0 palmitic (24.5%), and monounsaturated 16:1n-7 palmitoleic (27.2%) acids, whereas no polyunsaturated FA were encountered. Another informative link is provided by the comparison of our findings with the published data on pennate diatoms isolated from freshwater and brackish-water bodies. Freshwater strains *Gomphonema parvulum* SAG 1032-1 and *Navicula pelliculosa* SAG 1050-3 revealed a high content of saturated 16:0 palmitic acid (respectively, 36 and 15.5%) and monounsaturated 16:1n-7 palmitoleic acid (respectively, 31.1% and 50.2%) [70]. In addition, the *Gomphonema parvulum* SAG 1032-1 strain accumulated up to 8% of the long-chain polyunsaturated omega-6 22:4n-6 adrenic acid. A comparison of our findings with corresponding data on brackish-water strains reveals that, despite the similar prevalence of monounsaturated acids, the latter also accumulate long-chain polyunsaturated acids in considerable quantities. For instance, *Nitzschia frustulum* SAG 1052-52 profiles contained up to 23% of 16:1n-7 palmitoleic, up to 47.7% of 16:0 palmitic acids, and 14.3% of the polyunsaturated 22:4n-6 adrenic acid [70], whereas *Sellaphora pupula* FC2 profiles contained 29.3% of palmitoleic acid, 16.4% of palmitic acid, and 31.15% of the long-chain omega-6 20:4n-6 eicosatetraenoic acid [68]. It has been also demonstrated that *Phaeodactylum tricornutum* SAG 1090-1b is capable of accumulating higher concentrations of palmitoleic acid (up to 37.8% of total FA) at reduced content of palmitic acid (10.1%). A specific feature of marine strains of *Phaeodactylum tricornutum* is the high content of 20:5n-3 eicosapentaenoic acid (>23% of total FA) [70].

Altogether these lines of evidence suggest that FA repertoires in microalgae substantively depend on the habitat. In soil diatom strains, FA repertoires are dominated by saturated and monounsaturated acids. The algae isolated from brackish-water habitats present with higher content of long-chain polyunsaturated FA, whereas freshwater strains accumulate both saturated/monounsaturated and polyunsaturated FA. At the same time, algal taxa at different levels may show priorities toward the accumulation of certain types of fatty acids [6]. Proper assessment of the biochemical conservatism in species and strains of *Pinnularia* will require dedicated research on fatty acid composition for these algae from different habitats. FA profiles of the studied strains are heavily dominated by saturated acids (with a maximum of 92.7% in *P. minigibba* VP284) or saturated/monounsaturated acids (with the highest total in *P. minigibba* VP284 and *P. vietnamogibba* VP294). It might be sensible, therefore, to consider *Pinnularia* strains as potential producers of 16:0 palmitic and 16:1n-7 palmitoleic acids used in the production of biofuels [71].

### 3.4. Description of New Species

**Figure 3:**
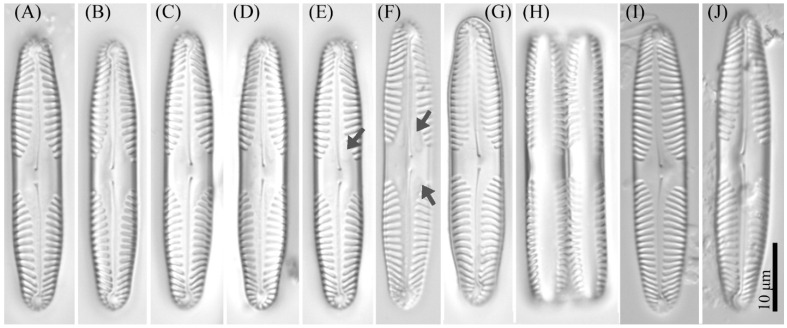
*Pinnularia minigibba* Kezlya, Maltsev, Krivova et Kulikovskiy sp. nov. Strain VP284, slide No. 07042. Light microscopy, differential interference contrast. (**A**–**G**,**I**,**J**) Valves face (arrows indicate the ghost striae). (**H**) Cell in girdle view. (**A**) Holotype. (**I**,**J**) Valves from the wild sample.

**Figure 4:**
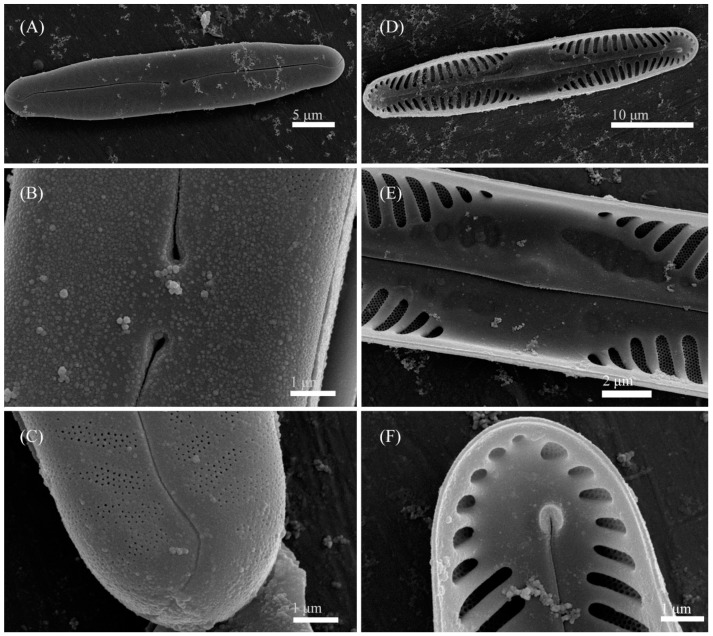
*Pinnularia minigibba* Kezlya, Maltsev, Krivova et Kulikovskiy sp. nov. Strain VP284, sample No. 07042. Scanning electron microscopy. (**A**–**C**) External views. (**D**–**F**) Internal views. (**A**,**D**) The whole valve. (**B**,**E**) Central are. (**C**,**F**) Valves ends. Arrows indicate the ghost striae.

***Pinnularia minigibba*** Kezlya, Maltsev, Krivova et Kulikovskiy sp. nov. (Figure 3 and Figure 4).

Diagnosis: Valves linear, sides slightly concave in the middle, length 40–43 μm, width 7–8 μm, length-to-width about 5.3–5.8, apices subcapitate, bluntly wedge-shaped to rounded width 4.8–5.5 μm (Figure 3). Raphe lateral, outer fissure straight or very weakly curved, central pores very small, slightly unilaterally deflected, drop-shaped, terminal fissures difficult to resolve, sickle-shaped, in the very small terminal area surrounded by striae at the poles. The axial area narrows, to 1/4 the width of the valve, continuously widening from the ends to the central area. Central area large, rhombic with a broad slightly asymmetric fascia, accompanied by ghost striae irregular in a shape, usually larger in the ventral side. Sometimes the ghost striae difficult to resolve in the LM. Striae radiate in the middle and convergent at the ends 9–10 in 10 μm.

Ultrastructure: In external views raphe branches straight (Figure 4A–C), slightly unilaterally deflected in the proximal part, terminated small, drop-shaped ends. The distal raphe ends are sickle-shaped and extend to the valve margin (Figure 4C). The striae are composed of one large alveolus, each alveolus is composed of five rows of small areolae.

In internal views (Figure 4D–F), the raphe is straight, proximal raphe endings are connected and represent a continuous slit, in the middle of the central area near a raphe is a well-developed unilaterally inflated central nodule. On either side of the central nodule are ghost striae (depths unequal, shapes irregular, more pronounced on the ventral side). Distal raphe ends straight, terminate on small helictoglossae. The alveoli are open.

Holotype: Slide no. 07042 (Holotype represented by Figure 3A) deposited at the collection of Maxim Kulikovskiy at the Herbarium of K.A. Timiryazev Institute of Plant Physiology RAS, 25 June 2019, collected by E.S. Gusev and E.M. Kezlya.

Reference strain: Strain VP284, isolated from soil sample KT54 deposited to the Algae Collection of Molecular Systematics of Aquatic Plants at K.A. Timiryazev Institute of Plant Physiology RAS.

Isotype: Slide no. 07042a, deposited in the collection of MHA, Main Botanical Garden RAS.

Sequence data: partial 18S rDNA gene sequence comprising V4 domain sequence (GenBank accession number OL739454) and partial *rbc*L sequence (GenBank accession number OL704397) for the strain VP284.

Type locality: Vietnam, Cát Tiên National Park, Đồng Nai Province, field soil, pH 4.91, the absolute humidity 24.68%, N11°23′35.8″ E107°21′9.48″.

Etymology: The specific epithet refers to the small dimensions of the valves and the name of a similar complex species *Pinnularia gibba*.

Distribution: As yet known only from the type locality.

**Figure 5:**
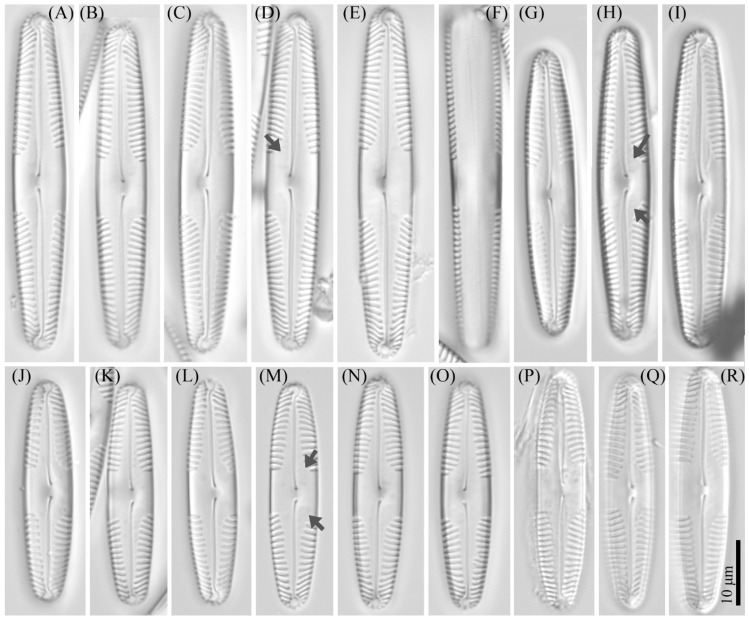
*Pinnularia vietnamogibba* Kezlya, Maltsev, Krivova et Kulikovskiy sp. nov. Strains VP294 slide No. 07052, VP290. slide No. 07048. Light microscopy, differential interference contrast. (**A**–**E**) Strain VP294, valve face (arrows indicate the ghost striae). (**J**–**O**) Strain VP290, valve face (arrows indicate the ghost striae). (**F**) Cell in girdle view. (**A**) Holotype. (**G**–**I**,**P**–**R**) Valves from the wild sample.

**Figure 6:**
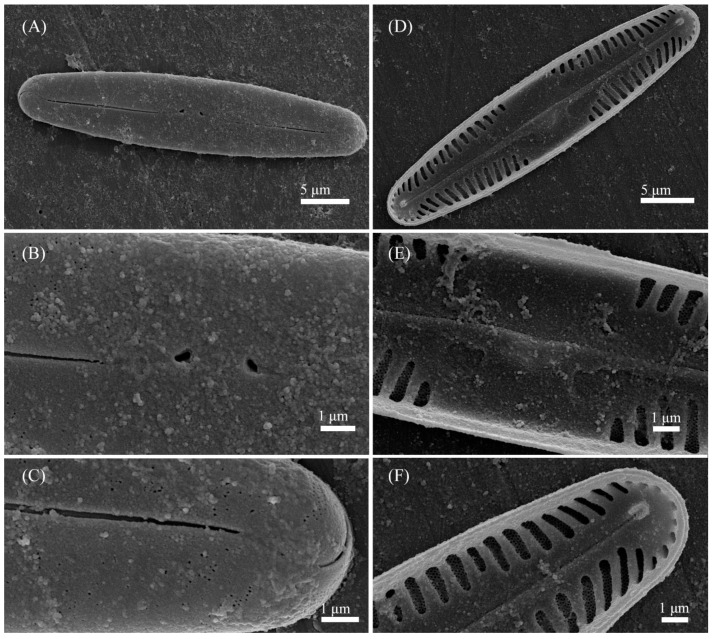
*Pinnularia vietnamogibba* Kezlya, Maltsev, Krivova & Kulikovskiy sp. nov. Strain VP294 sample No. 07052. Scanning electron microscopy. (**A**–**C**) External views. (**D**–**F**) Internal views. (**A**,**D**) The whole valve. (**B**,**E**) Central area. (**C**,**F**) Valves ends. Arrows indicate the ghost striae.

***Pinnularia vietnamogibba*** Kezlya, Maltsev, Krivova et Kulikovskiy sp. nov. (Figure 5 and Figure 6).

Diagnosis: Valves linear to linear elliptical with slightly convex or parallel sides, tapering to the broadly rounded apices, length 34–54 μm, width 7–8 μm, apices width 5 μm, length-to-width in small size valves about 4.7, in large size valves 6.4–6.75 (Figure 5). Raphe lateral, outer fissure straight, central pores very small, drop-shaped, slightly unilaterally deflected, terminal pores sickle-shaped. The axial area is moderately broad about 1/4 the width of the valve, linear or widening from the end to the central part of the valve, in small size valves lanceolate. Central area is large, rhombic with a broad slightly asymmetric fascia, accompanied by four ghost striae irregular in a shape, usually larger on the ventral side. Often the ghost striae are difficult to resolve in the LM. Striae radiate in the middle and strongly convergent at the ends 10–11 in 10 μm.

Ultrastructure: In external views raphe branches straight (Figure 6A–C), slightly unilaterally deflected in the proximal part, terminated small, drop-shaped ends. The distal raphe ends are sickle-shaped and extend to the valve margin (Figure 6C). The striae are composed of one large alveolus, each alveolus is composed of five rows of small areolae.

In internal views (Figure 6D–F), the raphe is straight, proximal raphe endings are connected and represent a continuous slit, in the middle of the central area near a raphe is a well-developed unilaterally inflated central nodule (Figure 6E). On either side of the central nodule are ghost striae unequal, irregular in the shape, larger on the ventral side. Distal raphe ends straight, terminate on small helictoglossae (Figure 6F). The alveoli are open.

Holotype: Slide no. 07052 (Holotype represented by Figure 5A) deposited at the collection of Maxim Kulikovskiy at the Herbarium of K.A. Timiryazev Institute of Plant Physiology RAS, 25 June 2019, collected by E.S. Gusev and E.M. Kezlya.

Reference strains: Strains VP290, VP294, isolated from soil sample KT61 deposited to the Algae Collection of Molecular Systematics of Aquatic Plants at K.A. Timiryazev Institute of Plant Physiology RAS.

Isotype: Slide no. 07052a, deposited in the collection of MHA, Main Botanical Garden RAS.

Sequence data: partial 18S rDNA gene sequences comprising V4 domain sequence (GenBank accession numbers: OL739456 for VP290, OL739458 for VP294) and partial *rbc*L sequences (GenBank accession numbers: OL704399 for VP290, OL704401 for VP294).

Type locality: Vietnam, Cát Tiên National Park, Đồng Nai Province, dry swamp soil, pH 5.1, the absolute humidity 49.02%, N11°24′24.7″ E107°23′6.61″.

Etymology: The specific epithet refers to the name of the country (Vietnam) where this species comes from and the name of a related complex species *Pinnularia gibba*.

Distribution: As yet known only from the type locality.

**Figure 7:**
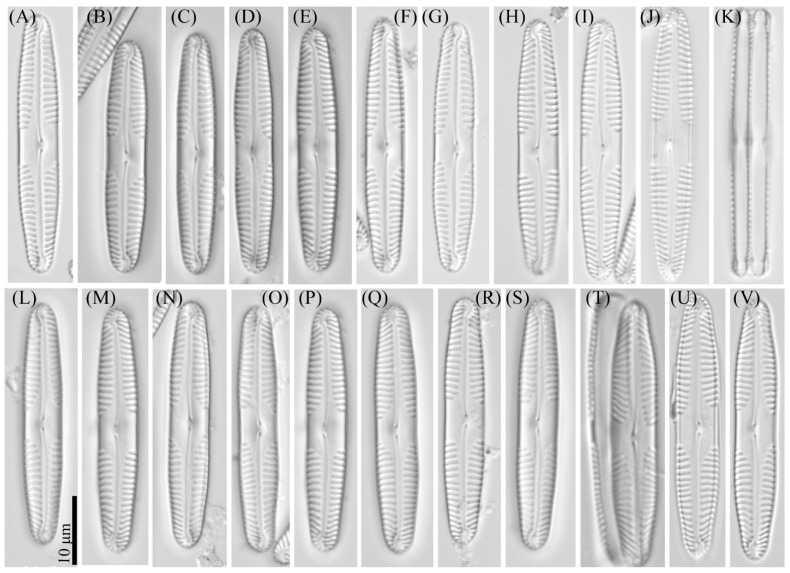
*Pinnularia microgibba* Kezlya, Maltsev, Krivova et Kulikovskiy sp. nov. Strains VP289 slide No. 07047, VP292. slide No. 07050. Light microscopy, differential interference contrast. (**A**–**J**) Strains VP289 valve face (arrows indicate the ghost striae). (**A**) Holotype. (**L**–**S**) Strain VP290 valve face (arrows indicate the ghost striae). (**K**) Cell in girdle view. (**T**–**V**) Valves from the wild sample.

**Figure 8:**
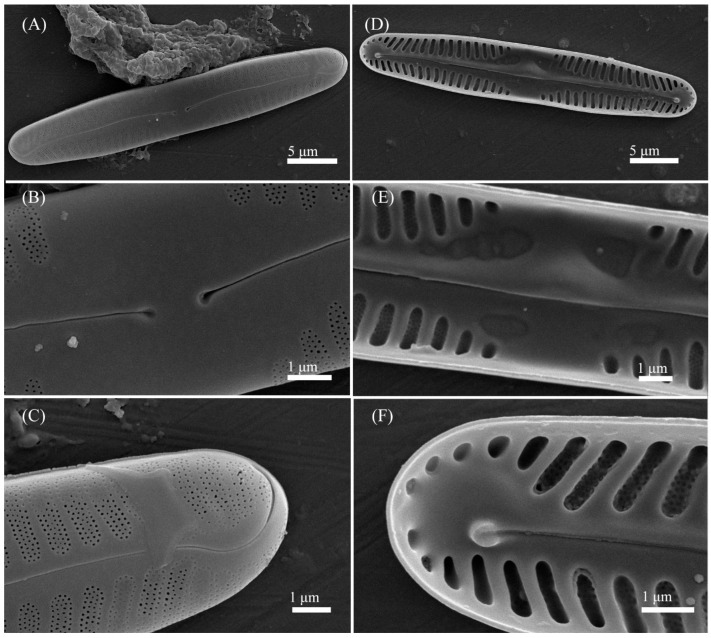
*Pinnularia microgibba* Kezlya, Maltsev, Krivova et Kulikovskiy sp. nov. Strain VP289 sample No. 07047. Scanning electron microscopy. (**A**–**C**) External views. (**D**–**F**) Internal views. (**A**,**D**) The whole valve. (**B**,**E**) Central area. (**C**,**F**) Valves ends. Arrows indicate the ghost striae.

***Pinnularia microgibba*** Kezlya, Maltsev, Krivova et Kulikovskiy sp. nov. (Figure 7 and Figure 8).

Diagnosis: Valves narrow-linear, sides slightly concave in the middle, length 35–40 μm, width 5.5–6.0 μm, length-to-width about 6.5–6.6, apices subcapitate, bluntly wedge-shaped to broadly rounded, slightly smaller valve width 3.8–4.5 μm (Figure 7). Raphe lateral, outer fissure straight or very weakly curved, central pores very small, slightly unilaterally deflected, drop-shaped, terminal fissures sickle-shaped. Axial area narrow, linear, slightly widening to the central part of the valve. Central area large, rhombic with a broad slightly asymmetric fascia, accompanied by four ghost striae irregular in a shape, usually larger on the ventral side. Sometimes the ghost striae are difficult to resolve in the LM. Striae parallel or slightly radiate in the middle and convergent at the ends 11–12 in 10 μm.

Ultrastructure: In external views raphe branches straight (Figure 8A–C), slightly unilaterally deflected in the proximal part, terminated small, drop-shaped ends. The distal raphe ends are sickle-shaped and extend to the valve margin (Figure 8C). The striae are composed of one large alveolus, each alveolus is composed of five rows of small areolae.

In internal views (Figure 8D–F), the raphe is straight, proximal raphe endings are connected and represent a continuous slit, with a well-developed unilaterally inflated central nodule in the middle of the central area. On either side of the central nodule are hollowed areas unequal, irregular in the shape, larger on the ventral side (Figure 8E). Distal raphe ends straight, terminate on small helictoglossae (Figure 8F). The alveoli are open.

Holotype: Slide no. 07047 (Holotype represented by Figure 7A) deposited at the collection of Maxim Kulikovskiy at the Herbarium of K.A. Timiryazev Institute of Plant Physiology RAS, 25 June 2019, collected by E.S. Gusev and E.M. Kezlya.

Reference strains: Strains VP289, and VP292, isolated from soil sample KT61 deposited to the Algae Collection of Molecular Systematics of Aquatic Plants at K.A. Timiryazev Institute of Plant Physiology RAS.

Isotype: Slide no. 07047a, deposited in the collection of MHA, Main Botanical Garden RAS.

Sequence data: partial 18S rDNA gene sequences comprising V4 domain sequence (GenBank accession numbers: OL739455 for VP289, OL739457 for VP292) and partial *rbc*L sequences (GenBank accession numbers: OL704398 for VP289, OL704400 for VP292).

Type locality: Vietnam, Cát Tiên National Park, Đồng Nai Province, dry swamp soil, pH 5.1, the absolute humidity 49.02%, N11°24′24.7″ E107°23′6.61″.

Etymology: The specific epithet refers to particularly small dimensions of the valves (micro-) and the name of a similar complex species *Pinnularia gibba*.

Distribution: Known from the type locality and samples KT39, KT80 (Table 1).

**Figure 9:**
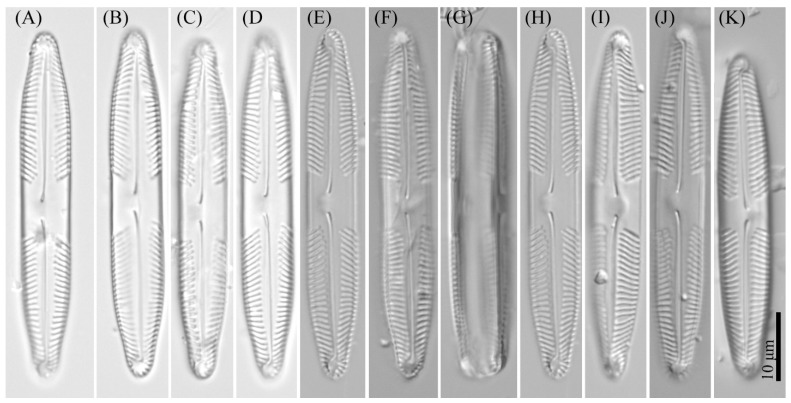
*Pinnularia insolita* Kezlya, Maltsev, Krivova et Kulikovskiy sp. nov. Strain VP280 slide No. 07038. Light microscopy, differential interference contrast. (**A**–**F**) Valve face. (**A**) Holotype. (**G**) Cell in girdle view. (**H**–**K**) Valves from the wild sample.

**Figure 10:**
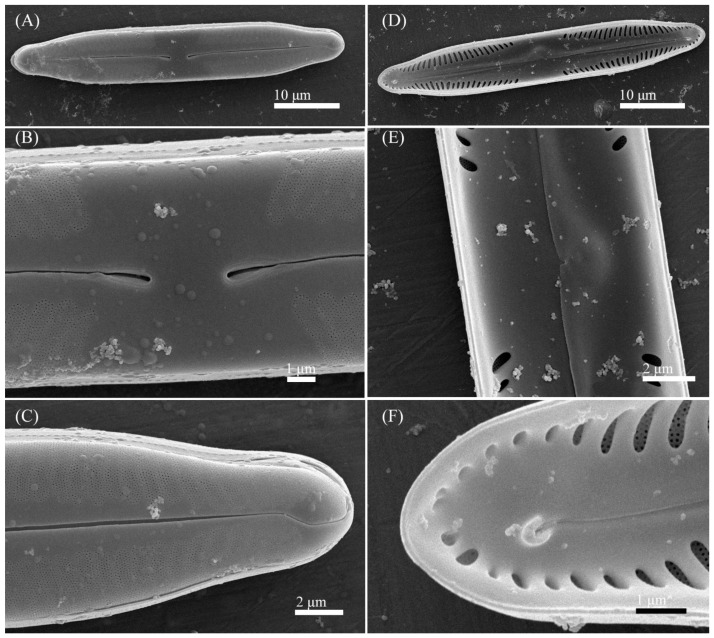
*Pinnularia insolita* Kezlya, Maltsev, Krivova et Kulikovskiy sp. nov. Strain VP280 sample No. 07038. Scanning electron microscopy. (**A**–**C**) External views. (**D**–**F**) Internal views. (**A**,**D**) The whole valve. (**B**,**E**) Central area. (**C**,**F**) Valves ends.

***Pinnularia insolita*** Kezlya, Maltsev, Krivova et Kulikovskiy sp. nov. (Figure 9 and Figure 10).

Diagnosis: Valves linear, sides slightly concave in the middle, length 50–52 μm, width 7–7.5 μm, length-to-width about 6.8–7.3, apices relatively long rostrate, finally broadly rounded width 4 μm (Figure 9). Raphe lateral, outer fissure straight, central pores relatively large, slightly unilaterally deflected, drop-shaped, terminal fissures not distinct, in the very small terminal area surrounded by striae at the poles. Axial area moderately broad, to 1/3 the width of the valve, widening from the end to the central part of the valve. The central area is very large with a broad slightly asymmetric fascia widening towards the valve margin, always bigger than the width of the valve. Striae radiate in the middle and strongly convergent at the ends 11–12 in 10 μm.

Ultrastructure: In external views raphe branches straight (Figure 10A–C). Proximal raphe ends are relatively long, drop-shaped, slightly unilaterally deflected. The distal raphe ends are sickle-shaped and extend to the valve margin (Figure 10C). The striae are very closely spaced and composed of one large alveolus; each alveolus is composed of six to seven rows of small areolae.

In internal views (Figure 10D–F) the raphe is straight, proximal raphe endings are connected and represent a continuous slit, terminating near a base well-developed unilaterally inflated central nodule. Distal raphe ends slightly deflected to one side and terminate on small helictoglossae (Figure 10F). The alveoli are open.

Holotype: Slide no. 07038 (Holotype represented by Figure 9A) deposited at the collection of Maxim Kulikovskiy at the Herbarium of K.A. Timiryazev Institute of Plant Physiology RAS, 25 June 2019, collected by E.S. Gusev and E.M. Kezlya.

Reference strain: Strains VP280 isolated from soil sample KT55 deposited to the Algae Collection of Molecular Systematics of Aquatic Plants at K.A. Timiryazev Institute of Plant Physiology RAS.

Isotype: Slide no. 07038a, deposited in the collection of MHA, Main Botanical Garden RAS.

Sequence data: partial 18S rDNA gene sequence comprising V4 domain sequence (GenBank accession number OL739453) and partial *rbc*L sequence (GenBank accession number OL704396) for the strain VP280.

Type locality: Vietnam, Cát Tiên National Park, Đồng Nai Province, forest soil, pH 5.55, the absolute humidity 44.8%, N11°23′9.17″ E107°22′52.3″.

Etymology: The specific epithet reflects the unusual shapes of the valves with narrowed apices and the central area.

Distribution: As yet known only from the type locality.

**Figure 11:**
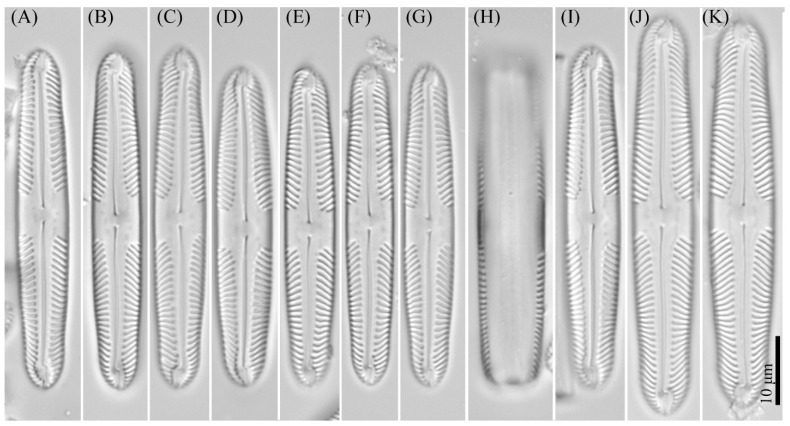
*Pinnularia ministomatophora* Kezlya, Maltsev, Krivova et Kulikovskiy sp. nov. Strain VP236 slide No. 06994. Light microscopy, differential interference contrast. (**A**–**G**) Valve face. (**A**) Holotype. (**H**) Cell in girdle view. (**I**–**K**) Valves from the wild sample.

**Figure 12:**
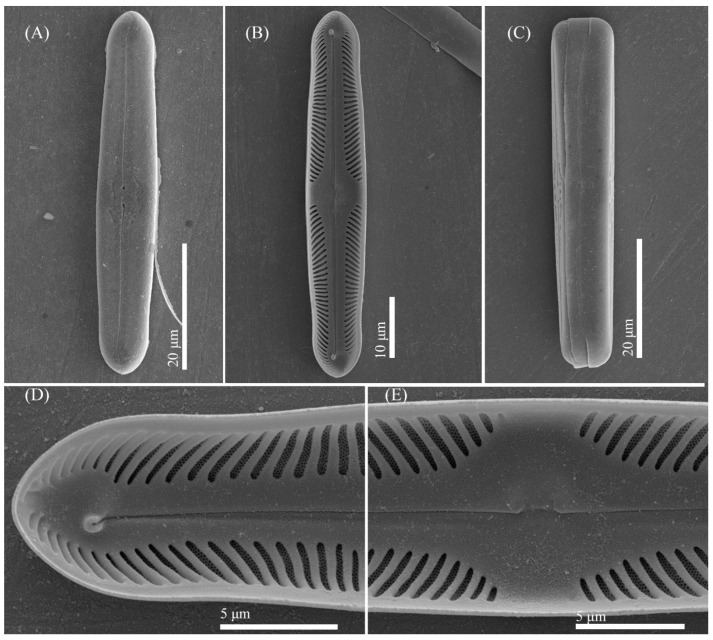
*Pinnularia ministomatophora* Kezlya, Maltsev, Krivova et Kulikovskiy sp. nov. Strain VP236 sample No. 06994. Scanning electron microscopy. (**A**) Exterior of the valve with the grooves on both sides of the raphe. (**B**,**D**,**E**) Internal views. (**C**) Cell in girdle view. (**E**) Central area. (**D**) Valves ends face.

***Pinnularia ministomatophora*** Kezlya, Maltsev, Krivova et Kulikovskiy sp. nov. (Figure 11 and Figure 12).

Diagnosis: Valves are linear in outline, sides slightly convex to parallel, apices broadly rostrate to broadly rounded, subcapitate, length 44–57 μm, width 7–9.5 μm, length-to-width about 5.9–6.7 (Figure 11). Raphe lateral, outer fissure straight or slightly broadly curved, central pores small, drop-shaped, straight or slightly curved on one side, terminal fissures long, bayonet shaped, lying in an elongate-elliptic terminal area. Axial area narrow, up to ¼ the width of the valve, widened from the ends to the rhombic central area which is differentiated in the valve middle with a broad symmetric or slightly asymmetric fascia. On either side of the central nodule in the central area are hollows in the valve surface irregular in the shape (Figure 11A–G,I–K and Figure 12A), in some valves, the hollows are difficult to resolve in LM. Striae 10–11 in 10 μm, strongly radiate in the middle and strongly convergent at the ends.

Ultrastructure: In external views the raphe is straight. Proximal raphe ends are drop-shaped, slightly unilaterally deflected. The distal raphe ends are sickle-shaped and extend to the valve margin (Figure 12A). The striae are very closely spaced and composed of one large alveolus; each alveolus is composed of five to seven rows of small areolae. In the central area on either side of the central nodule are hollows in the valve surface irregular in the shape.

In internal views (Figure 12B,D,E) the raphe is straight. The raphe branches are straight with short, bent, proximal raphe endings, terminating on a bad-developed unilaterally inflated central nodule. Distal raphe ends slightly deflected to one side and terminate on small helictoglossae (Figure 12D). The alveoli are open.

Holotype: Slide no. 07116 (Holotype represented by Figure 11A) deposited at the collection of Maxim Kulikovskiy at the Herbarium of K.A. Timiryazev Institute of Plant Physiology RAS, 3 June 2019, collected by E.S. Gusev and E.M. Kezlya.

Reference strain: Strain VP563 isolated from soil sample KT39 deposited to the Algae Collection of Molecular Systematics of Aquatic Plants at K.A. Timiryazev Institute of Plant Physiology RAS.

Isotype: Slide no. 07116a, deposited in the collection of MHA, Main Botanical Garden RAS.

Sequence data: partial 18S rDNA gene sequence comprising V4 domain sequence (GenBank accession number OL739459) and partial *rbc*L sequence (GenBank accession number OL704402) for the strain VP563.

Type locality: Vietnam, Cát Tiên National Park, Đồng Nai Province, soil from the bottom of a dry stream, pH 5.19 the absolute humidity 36.79%, N11°26′7.52″ E107°23′17.2″.

Etymology: The specific epithet refers to the small dimensions of the valves (mini-) and the name of a similar species *Pinnularia stomatophora*.

Distribution: Known from the type locality and sample KT70 (Table 1).

**Figure 13:**
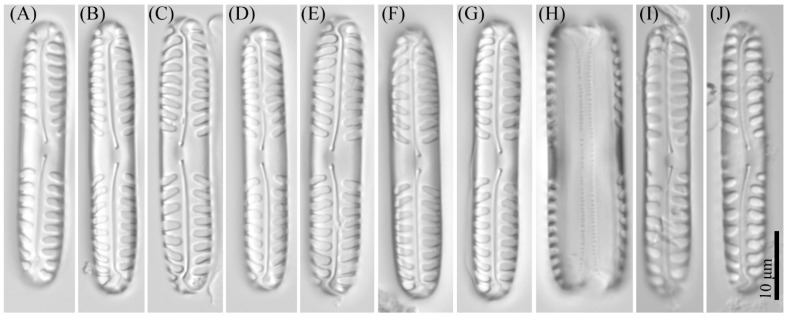
*Pinnularia paradubitabilis* Kezlya, Maltsev, Krivova et Kulikovskiy sp. nov. Strains VP563 slide No. 07116. Light microscopy, differential interference contrast. (**A**–**G**) Valve face. (**A**) Holotype. (**H**) Cell in girdle view. (**I**–**K**) Valves from the wild sample.

**Figure 14:**
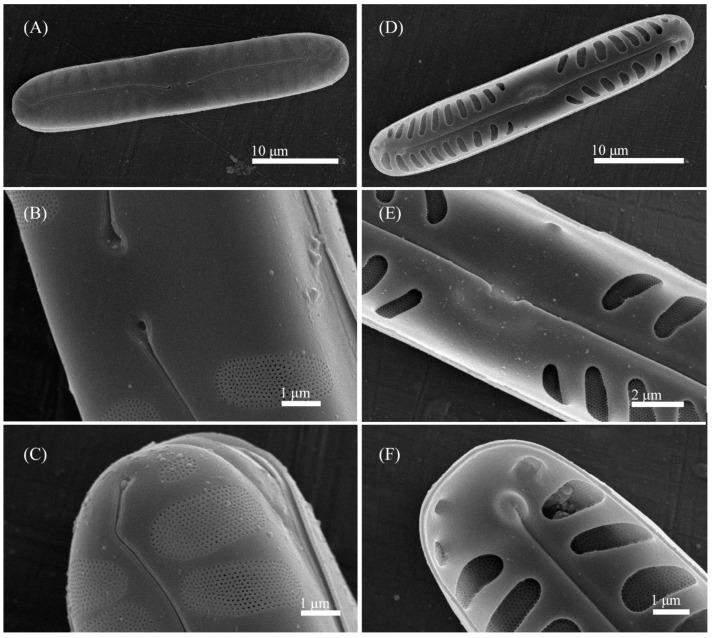
*Pinnularia paradubitabilis* Kezlya, Maltsev, Krivova et Kulikovskiy sp. nov. Strain VP563 sample No. 07116. Scanning electron microscopy. (**A**–**C**) External views. (**D**–**F**) Internal views. (**A**,**D**) The whole valve. (**B**,**E**) Central area. (**C**,**F**) Valves ends.

***Pinnularia paradubitabilis*** Kezlya, Maltsev, Krivova et Kulikovskiy sp. nov. (Figure 13 and Figure 14).

Diagnosis: Valves outline linear, margins parallel or slightly concave, apices bluntly rounded, length 39–41 μm, width 6–7 μm (Figure 13). Raphe moderately lateral the outer fissures straight to weakly curved, central pores small, drop-shaped, slightly curved on one side, terminal fissures long, sickle-shaped. Axial area narrow, linear, central area broad, more than the width of valve forming a broad fascia. Striae 5–6 in 10 μm, radiate or parallel in the middle, convergent (or sometimes parallel) at the ends.

Ultrastructure: In external views, the raphe branches are straight or broadly rounded, clearly unilaterally deflected in the proximal part (Figure 14A–C). Proximal raphe ends are drop-shaped. The distal raphe ends are sickle-shaped and extend to the valve margin (Figure 14C). The striae are composed of one large alveolus; each alveolus is composed of 12 to 15 rows of small areolae. The striae continued shortly on the valve margins. In internal views (Figure 14D–F) the raphe straight, proximal raphe branches are straight or short bent, terminating on a well-developed unilaterally inflated central nodule. Distal raphe ends slightly deflected to one side and terminate on small helictoglossae (Figure 14F). The alveoli are open.

Holotype: Slide no. 06994 (Holotype represented by Figure 13A) deposited at the collection of Maxim Kulikovskiy at the Herbarium of K.A. Timiryazev Institute of Plant Physiology RAS, 16 June 2019, collected by E.S. Gusev and E.M. Kezlya.

Reference strain: Strains VP236 isolated from soil sample KT53 deposited to the Algae Collection of Molecular Systematics of Aquatic Plants at K.A. Timiryazev Institute of Plant Physiology RAS.

Isotype: Slide no. 06994a, deposited in the collection of MHA, Main Botanical Garden RAS.

Sequence data: partial 18S rDNA gene sequence comprising V4 domain sequence (GenBank accession number OL739452) and partial *rbc*L sequence (GenBank accession number OL704395) for the strain VP236.

Type locality: Vietnam, Cát Tiên National Park, Đồng Nai Province, the surface of basalt in the forest, N11°26′9.75″ E107°21′46.2″. 

Etymology: The specific epithet refers to the resemblance to *Pinnularia dubitabilis* Hustedt. and name of country, when the species were found.

Distribution: Known from the type locality and samples KT19, KT 40, 974, 965 (Table 1). This species was identified by Niels Foged (p. 355, pl. XII, Figures 18 and 19 of [72]) as *P. borealis* Ehr. in a freshwater material from North Thailand (samples Loc. No. 9: Oblung Park (Hod District), ca 100 km SW of Chiengmai. 4/6 1966; Loc. No. 10: Mae Klong Water Fall (Chiengmai). 5/6 1966).

## 4. Conclusions

Investigations of diatom soil algae using molecular methods are still very limited. Using a polyphasic approach, we have described six new *Pinnularia* species from soils from an understudied region. Phylogenetic analysis based on 18S rDNA and *rbc*L genetic markers indicate that the species were from separate lineages. Based on unclear morphological differences, the conclusion was drawn that *P*. *microgibba* sp. nov. and *P*. *minigibba* sp. nov, *Pinnularia* sp. 6 (Tor4)r and *Pinnularia* sp. 3 (Tor8)b are pseudocryptic taxa. Analyses of morphological and molecular data have shown that the presence of ultrastructural surface markings in the central area consistently follows phylogenetic clades. The markings are an important morphological feature and require examination with a scanning electron microscope. The fatty acid composition of the cells of the studied soil *Pinnularia* strains showed an increased content of stearic, palmitic and palmitoleic fatty acids compared with brackish-water and freshwater species.

## Figures and Tables

**Table 1:** The geographic position of samples and measured ecological parameters.

Sample ID	Collection Date	Sample Locality	Habitat Type	Substratum	pH	Absolute Humidity, %	Species, Strains
KT19	5 June 2019	N11°26′8.13″ E107°26′42.9″ Cát Tiên National Park, Vietnam	forest	soil	5.0	21.32	*P. paradubitabilis*
KT53	16 June 2019	N11°26′9.75″ E107°21′46.2″ Cát Tiên National Park, Vietnam	forest	basalt	n.a.	n.a.	*P. paradubitabilis*, VP236
KT68	19 March 2020	N11°26′63.1″ E107°23′52.2″ Cát Tiên National Park, Vietnam	forest	soil	5.4	n.a.	*P. paradubitabilis*
965 975	23 November 2011	N18°36′8.08″ E102°24′6.05″ Vientiane province, Vang Vieng region, Nam Lik village, Laos	Nam Lik river	benthos periphyton	n.a.	n.a.	*P. paradubitabilis*
KT80	19 March 2020	N11°27′48.3″ E107°20′8.27″ Cát Tiên National Park, Vietnam	forest	soil	n.a.	n.a.	*P. microgibba*
KT39	7 June 2019	N11°26′7.52″ E107°23′17.2″ Cát Tiên National Park, Vietnam	forest, the bottom of a dry reservoir	soil	5.19	36.79	*P. microgibba* *P. ministomatophora*, VP563
KT61	25 June 2019	N11°24′24.7″ E107°23′6.61″ Cát Tiên National Park, Vietnam	dry swamp	soil	5.11	49.02	*P. microgibba*, VP289, VP292 *P. vietnamogibba*, VP290, VP294
KT70	19 March 2020	N11°26′6.60″ E107°23′7.39″ Cát Tiên National Park, Vietnam	forest, the bottom of a dry reservoir	soil	4.7	n.a.	*P. ministomatophora*
KT54	25 June 2019	N11°23′35.8″ E107°21′9.48″ Cát Tiên National Park, Vietnam	agricultural field	soil	4.91	24.68	*P. minigibba*, VP284
KT55	25 June 2019	N11°23′9.17″ E107°22′52.3″ Cát Tiên National Park, Vietnam	forest	soil	5.55	44.8	*P. insolita*, VP280

**Table 2:** Comparison of species and strains of *Pinnularia* from studied material with similar taxa.

Species, Strains	Outline, Apices	Valve Length, μm	Valve Width, μm	Number of Striae in 10 μm	Axial Area	Central Area	Markings on The Central Area	References
*P. minigibba* Kezlya, Maltsev, Krivova et Kulikovskiy, VP284	linear, slightly concave, apices subcapitate	40–43	7–8	9–10	narrow, linear, to 1/4 the breadth of valve	large, rhombic with a broad slightly asymmetric fascia	internally: ghost striae irregular in the shape	this study
*P. vietnamogibba* Kezlya, Maltsev, Krivova et Kulikovskiy, VP290, VP294	linear, linear-elliptical, slightly convex with broadly rounded apices	34–54	7–8.5	10–11	moderately broad about 1/4 the breadth of valve	large, rhombic with a broad slightly asymmetric fascia	internally: ghost striae irregular in the shape	this study
*P. microgibba* Kezlya, Maltsev, Krivova et Kulikovskiy, VP289, VP292	narrow-linear, slightly concave, apices subcapitate	35–40	5.5–6.0	11–12	narrow, linear	large, rhombic with a broad slightly asymmetric fascia	internally: ghost striae irregular in the shape	this study
*P. insolita* Kezlya, Maltsev, Krivova et Kulikovskiy, VP280	linear, slightly concave, apices rostrate	50–52	7–7.5	11–12	moderately broad, to 1/3 the breadth of valve	very large with a broad slightly asymmetric fascia widening towards the valve margin	no	this study
*P. ministomatophora* Kezlya, Maltsev, Krivova et Kulikovskiy, VP563	linear, slightly convex, apices subcapitate	44–57	7–9.5	10–11	narrow, up to 1/4 the breadth of valve	large with a broad slightly asymmetric fascia widening towards the valve margin	externally: hollows in the valve surface irregular in the shape	this study
*P. paradubitabilis* Kezlya, Maltsev, Krivova et Kulikovskiy, VP236	linear with parallel or slightly concave margins, apices obtusely rounded	39–44	6–7	5–6	narrow, linear	large with a broad slightly asymmetric fascia widening towards the valve margin	no	this study
*P. australogibba* var. *subcapitata* Van de Vijver, Chattová et Metzeltin	lanceolate to narrowly lanceolate with subcapitate apices	22–45	5.3–7.3	11–12	moderately broad, lanceolate,	large, rhombic–rounded, forming a broad fascia	internally: ghost striae	[55]
*P. parvulissima* Krammer	linear, slightly convex, apices very broadly rostrate to subcapitate and broadly rounded	34–70	10–12	8–10	1/4–1/3 the valve breadth	with a moderately broad slightly asymmetric fascia	internally: ghost striae, four large markings, larger in the ventral side	[11]
*P. microstauron* var. *angusta* Krammer	linear, apices always distinctly offset and much smaller than the valve width, waged-shaped	25–47	6.5–8.0	10–12	narrow, linear	with a broad slightly asymmetric fascia	no date, not visible on LM photo	[11,57,61]
*P. gibba* var. *subsancta* Manguin	linear-lanceolate with hardly protracted apices	37.5	7.5–8.0	13–15	very broad, lanceolate	rectangular with a fascia	internally: ghost striae	[11,58,59]
*P. australogibba* Van de Vijver, Chattová et Metzeltin	lanceolate to narrowly lanceolate, weakly protracted, rostrate, broadly rounded apices	45–60	7.8–9.4	12–13	moderately broad, lanceolate	large, rhombic–rounded, forming a broad fascia	internally: ghost striae	[55]
*P. tagliaventiae* Lange-Bertalot et Metzeltin	strictly linear, slightly triangulate, apices broadly protracted, rounded to weakly cuneate	40–70	7–10	10–11	broad and widened deltoid towards the central area	with a broad fascia over the valve face and mantle	no date, not visible on LM photo	[62]
*Pinnularia* sp. Tor4r	narrow-linear, slightly concave, apices subcapitate	42.6 ± 0.4	5.8 ± 0.3	12.4 ± 0.5	narrow, linear *	large with a broad slightly asymmetric fascia widening towards the valve margin *	no date, not visible on LM photo	[25]
*Pinnularia* sp. Tor8b	narrow-linear, slightly concave, apices subcapitate	40 ± 0.5	5.9 ± 0.3	11.8 ± 0.3	narrow, linear *	large with a broad slightly asymmetric fascia widening towards the valve margin *	internally: ghost striae *	[25]
*P. sinistra* Krammer	linear, slightly convex or concave, apices indistinctly differentiated, broadly protracted	17–52	4–6.5	11–14	linear, in large individuals lanceolate	slightly asymmetric fascia	no	[11,55,63]
*P. subcapitata* W.Gregory (given as *P. hilseana* Janisch)	linear to weakly linear-elliptical, apices distinctly offset capitate	17–57	4–6.8	10–14	linear to narrowly lanceolate, expanding into a fascia	fascia	no date, not visible on LM photo	[64]
*P. saprophila* Lange-Bertalot, Kobayasi et Krammer	linear, sides weakly convex, apices distinctly offset, capitate, in small individuals subcapitate or broadly protracted	21–45	5.7–7.5	9.5–11	lanceolate	large, rhombic with a broad fascia	no date, not visible on LM photo	[11]
*P. pisciculus* Ehrenberg	linear, sides straight to very weakly convex or concave to triangulate, apices capitate	22–50	6.0–8.3	10.5–12	narrow or lanceolate	relatively large, rhombic, widened into fascia	no date, not visible on LM photo	[11,65]
*P. similiformis* var. *koreana* Metzeltin et Krammer	linear, linear-lanceolate to rhombic-lanceolate, apices not offset, obtusely cuneate-rounded	40–60	7.7–8.0	10–12	very narrow, to 1/5 of valve width, linear to slightly lanceolate	rhombic, widened into broad fascia	no date, not visible on LM photo	[11]
*P. marchica* I. Schönfelder	linear to elliptic-lanceolate, in larger specimens slightly concave, apices relatively long rostrate or subcapitate	22–37	4.7–6.3	11–14	narrow, linear	broad rhombic fascia	no date, not visible on LM photo	[11]
*P. obscura* Krasske	linear-elliptical with straight to weakly convex or concave sides, apices weakly rostrate or cuneiform and not offset and broadly rounded	12–34	3–5.4	10–13	very narrow, linear	large, widened into fascia	no	[11,56,66]
*P. brebissonii* var. *bicuneata* Grunow	linear, sides almost straight and parallel, apices distinctly obtuse or acutely wedge-shaped	14–60	8–11	9–13	narrow, linear	broadly rhombic fascia	no date, not visible on LM photo	[11]
*P. cavancinii* Lange-Bertalot et Metzeltin	rhombic-lanceolate to elliptic-lanceolate, apices gently but distinctly protracted to a wadge, finally obtusely rounded	32–48	7.5–9.0	12–13	lanceolate	fascia, which is rather broad and extended over the mantle	no date, not visible on LM photo	[57]
*P. stomatophora* var. *irregularis* Krammer	linear, apices obtusely rounded	40–70	10–11	12–13	moderately broad, 1/4–1/3 the breadth of the valve, linear to linear-lanceolate	rectangular with a small fascia	externally, diverse structured flecks on both sides	[11]
*P. graciloides* var. *triundulata* (Fontell) Krammer	linear, sides slightly undulate, undulates in small valves nearly absent, apices broadly rounded	82–105	11–13	10–12	linear, 1/4–1/3 the breadth of the valve	rhombic, with broad fascia	externally: irregular markings, often difficult to see	[11]
*P. subgibba* var. *undulata* Krammer	linear, sides weakly undulate, apices slightly capitate	52–84	8–10	9–10	broader, almost 1/4–1/2 breadth of the valve	with broad fascia	internally: ghost striae irregular in the shape on both sides and larger on the ventral side	[11]
*P. borealis* Ehrenberg	linear, linear-elliptical, margins parallel to weakly convex, apices rounded	24–42	8.5–10	5–6	narrow	large, rounded, 1–2 central striae often absent	no	[1,11,28]
*P. angustiborealis* Krammer et Lange-Bertalot	linear, margins moderately convex, apices broadly subrostrate	34–45	7.4–8.0	5–6 (7 *)	moderately narrow, widening	transverse fascia	no date, not visible on LM photo	[11]
*P. dubitabilis* Hustedt	rectangular, linear, margins parallel, apices bluntly rounded	23–40	6–7	3–5	wide	absent or fascia	no date, not visible on LM photo	[11]
*P. intermedia* (Lagerstedt) Cleve	linear with straight to weakly convex sides, apices capitate or not, and broadly to obtusely rounded	(15)18–40	4.8–7	7–10	very narrow	moderately broad, widened into a fascia	no date, not visible on LM photo	[11]
*P. angulosa* Krammer	rectangular, linear, margins parallel, apices broadly rounded	42–53	9.7–10.3	3–4	wide	nearly absent	no date, not visible on LM photo	[11]

* counted from published data.

**Table 3:** Fatty acid composition of new *Pinnularia* strains, the data are reported as the mean (% of total fatty acids and mg g^−1^ dry biomass) ± standard error from three independent biological replicates.

Fatty Acid	*Pinnularia ministomatophora*	*Pinnularia vietnamogibba*		*Pinnularia minigibba*	*Pinnularia microgibba*		*Pinnularia insolita*	*Pinnularia paradubitabilis*
	VP563	VP290	VP294	VP284	VP292	VP289	VP280	VP236
anteiso-15:0 Sarcinic acid						0.7 ± 0.02		
10:0 Capric acid		0.3 ± 0.01						
12:0 Lauric acid	0.3 ± 0.01		0.2 ± 0.01	0.2 ± 0.02	0.2 ± 0.01	0.4 ± 0.01	0.2 ± 0.02	0.2 ± 0.01
14:0 Myristic acid	4.6 ± 0.1	3.1 ± 0.04	4.0 ± 0.1	3.0 ± 0.1	6.3 ± 0.1	2.1 ± 0.1	5.1 ± 0.1	1.7 ± 0.04
16:0 Palmitic acid	30.4 ± 0.8	26.7 ± 0.6	26.8 ± 0.7	24.6 ± 0.7	30.3 ± 0.8	20.1 ± 0.52	23.4 ± 0.7	25.4 ± 0.5
18:0 Stearic acid	45.7 ± 1.3	58.2 ± 1.7	51.3 ± 1.7	64.4 ± 1.7	36.0 ± 0.7	61.1 ± 1.9	48.9 ± 1.8	50.2 ± 1.5
20:0 Arachidic acid	0.2 ± 0.01	0.3 ± 0.01	0.3 ± 0.01	0.3 ± 0.01	0.1 ± 0.01	0.4 ± 0.02	0.2 ± 0.01	0.2 ± 0.01
22:0 Behenic acid			0.1 ± 0.01	0.2 ± 0.01				0.1 ± 0.01
16:1n-7 cis-9-Palmitoleic acid	15.2 ± 0.7	9.7 ± 0.8	15.5 ± 0.5	5.5 ± 0.4	20.8 ± 0.6	2.7 ± 0.1	17.6 ± 0.8	15.3 ± 0.6
16:1n-5 cis-11-Palmitovaccenic acid								0.2 ± 0.01
18:1n-11 cis-7-Vaccenic acid								1.3 ± 0.1
18:1n-9 cis-9-Oleic acid	1.5 ± 0.04	1.7 ± 0.04	0.4 ± 0.02	1.3 ± 0.04	1.6 ± 0.1	6.4 ± 0.2	1.3 ± 0.02	1.9 ± 0.1
18:1n-7 cis-11-Vaccenic acid			0.7 ± 0.04					
16:2n-6 cis-7,10-Hexadecadienoic acid			0.3 ± 0.02					
16:2n-4 cis-9,12-Hexadecadienoic acid					2.4 ± 0.1			0.7 ± 0.02
18:2n-6 cis-9,12-Linoleic acid						5.5 ± 0.2	0.2 ± 0.02	1.1 ± 0.03
16:3n-4 cis-6,9,12-Hexadecatrienoic acid	1.1 ± 0.03						1.4 ± 0.1	0.9 ± 0.03
18:3n-6 cis-6,9,12-gamma-Linolenic acid			0.4 ± 0.02					0.8 ± 0.02
20:4n-6 cis-5,8,11,14-Arachidonic acid	1.0 ± 0.03			0.5 ± 0.02	2.3 ± 0.1	0.6 ± 0.02	0.6 ± 0.02	
20:5n-3 cis-5,8,11,14,17-Eicosapentaenoic acid							1.1 ± 0.03	
total SFAs	81.2 ± 2.3	88.6 ± 2.3	82.7 ± 2.5	92.7 ± 2.5	72.9 ± 1.6	84.8 ± 2.4	77.8 ± 2.6	77.8 ± 2.1
total MUFAs	16.7 ± 0.8	11.4 ± 0.8	16.6 ± 0.5	6.8 ± 0.4	22.4 ± 0.5	9.1 ± 0.3	18.9 ± 0.8	18.7 ± 0.8
total PUFAs	2.1 ± 0.1		0.7 ± 0.03	0.5 ± 0.02	4.7 ± 0.2	6.1 ± 0.2	3.3 ± 0.1	3.5 ± 0.1
total fatty acids, mg g^−1^ dry biomass	43.9 ± 2.3	44.3 ± 2.1	42.7 ± 1.9	47.8 ± 2.4	40.1 ± 1.8	39.5 ± 2.2	33.7 ± 1.8	27.8 ± 1.5

## Data Availability

Not applicable.

## Supplementary Materials

The following supporting information can be downloaded at: https://www.mdpi.com/article/10.3390/cells11152446/s1, Appendix S1 Alignment of the 18S rDNA gene partial sequence and *rbc*L gene, in txt format. Appendix S2 The Bayesian phylogenetic tree topology, in txt format.
